# Poison Ivy Dermatitis Treatment Patterns and Utilization: A Retrospective Claims-based Analysis

**DOI:** 10.5811/westjem.2022.March.55516

**Published:** 2022-06-30

**Authors:** Melissa Butt, Avram Flamm, James G. Marks, Alexandra Flamm

**Affiliations:** * Penn State Health, Department of Dermatology, Hershey, Pennsylvania; † Penn State College of Medicine, Department of Public Health Science, Hershey, Pennsylvania; ‡ Penn State Health, Department of Family and Community Medicine, Hershey, Pennsylvania; § Penn State Health, Department of Emergency Medicine, Hershey, Pennsylvania

## Abstract

**Introduction:**

Poison ivy (toxicodendron) dermatitis (TD) resulting from contact with poison ivy, oak, or sumac is a common form of allergic contact dermatitis that impacts millions of people in the United State every year and results in an estimated 43,000 emergency department (ED) visits annually. Our objective in this study was to evaluate whether healthcare utilization outcomes are impacted by prescription practices of systemic corticosteroids.

**Methods:**

We used a health claims database from 2017–2018 of those treated for TD. Descriptive statistics and logistics regression models were used to characterize trends.

**Results:**

We included in this analysis 115,885 claims from 108,111 unique individuals (93.29%) with 7,774 (6.71%) return claims within 28 days. Of the return claims, 470 (6.05%) were to the ED. Emergency clinicians offered no oral corticosteroid prescription 5.27% (n = 3,194) of the time; 3276 (86.26%) prescriptions were for a duration of 1–13 days, 410 (10.80%) were for 14–20 days, and 112 (2.95%) were for >21 days. Further, we found that shorter duration oral corticosteroids (odds ratio [OR] 1.30; 95% confidence interval 1.17–1.44; P <0.001) and initial treatment for TD at the ED compared to primary care clinicians (OR 0.87 [0.80, 0.96]; P <0.001) and other non-dermatologists (OR 0.89 [0.80, 0.98]; P = 0.01) places patients at an increased risk for return visits with healthcare clinicians when controlling for drug group, duration of treatment, and initial treatment location.

**Conclusion:**

Despite recommendations to treat TD with oral steroids for at least 14 days, most emergency clinicians offered this treatment for shorter durations and was associated with return visits. Emergency clinicians should consider treatment of two to three weeks when providing systemic steroid coverage when there are no limiting contraindications, especially as patients who present to the ED may do so with more severe disease. Additional education may be needed on appropriate treatment pathways for TD to reduce healthcare utilization associated with undertreatment.

## INTRODUCTION

Poison ivy (toxicodendron) dermatitis (TD) results from contact with poison ivy, oak, or sumac and is a common form of allergic contact dermatitis (ACD) that affects millions of people in the United States every year,^1^ and it accounts for an estimated 43,000 annual visits to the emergency department (ED). Due to seasonal effects, a number of states see an increase in the number of cases during the summer months, likely due to increases in the growth of the plants containing urushiol, the allergen causing TD, as well as the increase in the number of individuals participating in outdoor activities.^2^ While some cases of TD remain mild and can be managed at home with little to no medical intervention, other cases can elicit more severe reactions. Toxicodendron dermatitis can cause discomfort and marked itching, as well as the formation of blisters. Depending on the location of these eruptions, patients can also suffer from limitations in activities of daily living, such as sitting, walking, or mental concentration due to these symptoms.

Like other forms of ACD, the treatment of TD relies on the use of topical and/or systemic corticosteroids to suppress the immune response to urushiol. However, the strength and ideal duration of such pharmaceutical interventions is not well established in the literature. It has been demonstrated that treatment plans that are too short are less likely to be effective in controlling the symptoms. In particular, the effectiveness of short-course, prepackaged oral corticosteroids is of questionable use. Ives and Tepper reported a number of severe cases treated with prepackaged methylprednisolone in which the patients did not achieve effective control of their symptoms.^3^ Further, several papers have also cautioned against the use of prepackaged oral corticosteroids that provide a short duration of treatment, due to the risk of rebound dermatitis after shorter therapeutic interventions.^4^,^5^

Despite these recommendations, a recent study of healthcare claims revealed that there are variable uses of corticosteroids both in terms of potency and route of administration (eg, topical vs oral).^2^ The cost of these treatments varied depending on healthcare setting (eg, outpatient vs emergency) and type of treatment.^2^ The majority of these claims took place in a primary care setting, with 6% being seen in the ED or by emergency clinicians.^2^

Due to the variability in the treatments and healthcare setting, we hypothesized that this variability could result in the prescription of subtherapeutic therapies for patients presenting with TD, resulting in poorer health utilization outcomes, including increased risk of return visits. Thus, our objective in this study was to identify frequency patterns of various oral corticosteroid prescription durations and evaluate the impact of prescription duration on health utilization outcomes, particularly in terms of return visits within 30 days to the ED.

## METHODS

This study included a retrospective analysis of healthcare claims from the IBM MarketScan Research Databases (IBM Corporation, Armonk, NY). These databases contain de-identified healthcare claims from 2017–2018 for approximately 27 million privately insured individuals residing in the US. Those with Medicare or Medicaid are not included in this data and thus the sample is restricted to only those <65 years of age. Specific data abstracted for this analysis included basic demographic information along with details regarding the date, clinician type, and purpose of the visit for outpatient healthcare encounters. These databases also include details on prescription claims including the date of the claim, National Drug Code (NDC) numbers, refill counts, and days’ supply.

*Population Health Research Capsule*

What do we already know about this issue?

*Toxicodendron dermatitis (TD) is a common, seasonal dermatologic condition that affects millions of people in the United States annually.*

What was the research question?

*How does type and duration of treatment for TD impact odds of return healthcare visits within 28 days?*

What was the major finding of the study?

*Shorter duration (<14 days) of treatment is associated with 1.30 increased odds of a return healthcare visit.*

How does this improve population health?

*Providing adequate oral corticosteroid coverage can reduce healthcare utilization and cost of care for the treatment of TD.*

**Figure 1:**
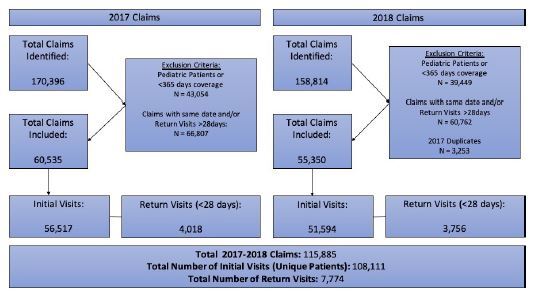
Flow diagram of claim selection and inclusion for patients treated for toxicodendron dermatitis in the United States.

We included only adult patients who had at least one outpatient claim for TD during the study period. No sample size calculation was conducted a priori as all eligible claims were included in the analysis. Outpatient healthcare encounter claims were identified by the *International Classifications of Disease 10^th^ Revision* (ICD-10) codes for ACD due to contact with plants (except food [L23.7]), which is largely due to TD. As patients can accrue multiple claims per day and contract TD multiple times per year, only one claim per day was included per patient and restricted to the first annual encounter. We also removed duplicate patient encounters in 2018 from the analysis to elininate the potential impact of patient-specific variation. A flow diagram of participant selection and inclusion is provided in Figure 1. Prescription treatments were restricted to oral systemic corticosteroids. In the study period we also evaluated follow-up treatment for 28 days after the first claim, and we identified return visits as those where the ICD-10 code for ACD due to plants was also used.

Claims were identified by place of service and divided into five categories based on current procedural terminology codes and MarketScan place of service identifiers: ED; urgent care (UC); dermatology; primary care physicians (PCP) including family and internal medicine clinicians; and other non-dermatology clinicians (such as non-specified nursing visits, geriatric medicine, allergy and immunology, etc). Oral corticosteroids were identified using NDC numbers and included dexamethasone, methylprednisolone, prednisolone and prednisone. Duration of treatment was also broken into four groups: no oral corticosteroid treatment; 1–13 days of treatment; 14–20 days; and 21 days or more based on the days’ supply.

### Statistical Analysis

Descriptive summary statistics were used to characterize overall trends in the data. We built univariable logistic regression models for dichotomous outcome variables predicting a return visit within 28 days and a return visit to the ED within the same time frame. Predictor variables included drug type, duration of treatment, and initial treatment location. Odds ratios (OR) were calculated with 95% confidence intervals (CI). We created an additional multivariable model using drug group, days’ supply, and initial location as predictors for a return healthcare visit. Due to limited occurrences, we excluded prednisolone and dexamethasone in these regression analyses.

To validate the data extraction methods and final univariable and multivariable models, we first conducted the analysis on the 2017 data and re-ran it using the 2018 data as an independent sample. Once the data extraction methods and models were verified and consistent across both years, the data were merged, and the final models were applied to the combined years of data after removing duplicates. Goodness-of-fit for logistic regression models was evaluated using the Hosmer-Lemeshow goodness-of-fit test via the LACKFIT option in SAS. Large values for the chi-square for Hosmer-Lemeshow (χ^2^_HL_) and small *P*-values (<0.05) were indicative of poor model fit. All final models used in this analysis failed to meet this criterion for poor fit and were therefore accepted as valid models. We conducted all statistical analyses using SAS version 9.4 (SAS Institute Inc, Cary, NC). This study was approved by the Penn State University Human Subjects Protection Program Institutional Review Board.

## RESULTS

### General Characteristics of Claims

During the study period, a total of 115,885 claims were identified and included in this analysis with 108,111 (93.29%) unique individuals who were seen for TD (characteristics of the claims are presented in Table 1). Nearly half of these patients were male (n = 56,002; 51.80%) with an average age of 44.19 years (standard deviation 13.17). The ED and UC visits made up 16.32% (n = 17,645) of the total initial visits, while PCPs made up the majority of clinicians for the initial visit with a total of 47,719 (44.14%). Non-dermatology clinicians contributed to 30.55% (n = 33,033) of initial visits while dermatologists made up 8.99% (n = 9,714). Within 28 days of the initial visit, an additional 7,774 (6.71%) patients incurred at least one return visit. Of these return visits, 470 (6.05%) were to an emergency clinician.

### Prescription Trends

Trends in oral corticosteroid prescriptions and treatments at the initial visit are presented in Table 2. In terms of treatment options, more than half of patients (56.09%; n = 60,637) received no oral corticosteroid as treatment for their TD and 42.94% were prescribed at the initial visit (n = 46,425). Of those with an oral corticosteroid prescription prescribed at the initial visit, 81.14% were for a supply of 1–13 days (n = 37,521), 16.59% were for a supply of 14–20 days (n = 7,672), and 2.27% were for a supply of **≥**21 days (n = 1,052). In terms of prescription duration and specialty, clinicians in the ED offered no oral corticosteroid prescription 5.27% (n = 3,194) of the time, and 86.26% prescriptions were for a duration of 1–13 days (n = 3,276). Prednisone made up the majority of first prescriptions (83.98%; n = 38,990), followed by methylprednisolone (15.25%; n = 7,078). Additionally, most methylprednisolone prescriptions (99.52%; n = 7,044) were for a duration of 1–13 days while only 77.75% (n = 30,315) of the prednisone prescriptions were for 1–13 days.

### Impact of Treatment on Healthcare Utilization

Table 3 shows factors associated with an increase in healthcare utilization. In terms of return visits within 28 days, receiving no prescription resulted in a lower likelihood of having a return visit both in terms of drug group and duration (OR 0.68 [0.65, 0.72] and OR 0.84 [0.76, 0.93], respectively). Those who received methylprednisolone had increased odds of a return visit when compared to those who received prednisone (OR 1.13 [1.02, 1.24]). Similarly, those who received a prescription for 1–13 days’ supply had increased odds of a return visit when compared to those who received a script for 14–20 days (OR 1.32 [1.19, 1.46]). Lastly, those first seen in the ED were also more likely to experience return visits as well as return visits to the ED when compared to all other specialists.

When we included and controlled for all variables, drug group, duration of treatment, and initial treatment location remained statistically significant predictors of a return healthcare visit (Table 4). Most notably, duration of treatment 1–13 days retained a significantly higher likelihood (OR 1.30 [1.17,1.44]) of a return healthcare visit compared to those treated for 14–20 days, after controlling for drug type and initial treatment location. Interestingly, we found no significant difference in the likelihood of a return healthcare visit for those initially seen in the ED when compared to those seen by dermatologists in the multivariable model.

## DISCUSSION

This study revealed that oral corticosteroids were prescribed to treat TD at the initial visit for less than half of visits. Most prescriptions were for durations of less than two weeks, which is shorter than the recommended treatment duration reported in the literature.^6^ As the immunologic response to urushiol can take up to 14 days to present in sensitized patients with exposure, TD reactions have the potential to continue to manifest or worsen throughout that 14-day period.^1^,^7^ Thus, shorter courses of oral corticosteroids pose the risk of patients experiencing rebound dermatitis,^1^,^4^ where signs and symptoms of an acute dermatitis can recur or flare after temporary suppression with an immunosuppressive medication, such as oral corticosteroids. This may result in the need for additional healthcare intervention due to its symptomatic nature. As oral corticosteroids are generally reserved and recommended for moderate to severe cases of TD, they should be prescribed for longer courses when medically indicated to prevent the possibility of rebound dermatitis.

Our findings further support and expand on previous clinical trial research. In 2014, Curtis and Lewis conducted a randomized controlled trial comparing a five-day course of prednisone to a 15-day tapered course of prednisone.^8^ Those receiving the longer course experienced improvement and resolution of symptoms sooner than those on the shortened course (approaching statistical significance) and used fewer supplementary medications such as prescription oral and topical corticosteroids as well as over-the-counter treatments (eg, calamine lotion, antihistamines, hydrocortisone cream, and other lotions).^8^ Additional publications have also supported that a minimum of 14–21 days of oral corticosteroid treatment is necessary when being prescribed for TD,^1^,^6^,^9^–^15^ as the hypersensitivity reaction can take up to 14 days to clinically manifest.

This study found that shorter duration oral corticosteroids can be problematic in the treatment of TD both independently and controlling for drug type and treatment location. As the majority of methylprednisolone prescriptions were for shorter duration courses, the use of methylprednisolone also became a significant predictor of return healthcare visits in the univariable analyses. However, when controlling for duration of treatment and initial treatment location, this association pulled closer to the null hypothesis and was no longer statistically significant suggesting that duration of treatment is driving this association. These findings also expand on the conclusions drawn by Curtis and Lewis that shorter duration oral corticosteroids can also result in higher odds of having a return visit, which could increase the cost of care for the patients.

Treatment in an ED was predictive of return visits within 28 days when compared to other clinician groups, except for dermatologists in the multivariable model. These findings of increased healthcare utilization are consistent with several studies showing that repeat utilization of the ED makes up for a large number of ED visits.^16^ The lack of difference in healthcare utilization outcomes between dermatologists and ED clinicians after controlling for drug type and duration of treatment could be explained by the severity of the patients seen in these two specialties. As dermatologists are typically not first-line clinicians who treat TD and EDs are generally used for more urgent health concerns, the potential presence of more severe cases in these two practices highlights the need for more clinical guidance regarding the appropriate treatment pathways for TD in an ED setting. These utilization patterns could also be the result of EDs commonly requesting patients to follow up with their PCP for their condition or could reflect more limited training in use of oral corticosteroids for TD and similar conditions; however, emergency clinicians did prescribe similar proportions of oral corticosteroids for 1–13 days as UC clinicians and PCPs. While emergency clinicians offered oral corticosteroids prescriptions for TD, many (86.26%) were for less than two-weeks duration. Less than 3% of emergency clinicians prescribed oral corticosteroids for more than 21 days, a treatment associated with higher efficacy and reduction in return visit rates. High ED utilization for the treatment of TD can also lead to increased healthcare costs as ED costs are higher overall.^16^ as well as for TD in particular.^1^,^2^

The findings also demonstrated that outcomes for those who received no prescription were better in terms of healthcare utilization (eg, return healthcare visits) compared to those who received treatment. This finding stands in contrast to our other findings. However, if those patients receiving no prescription were a patient population with very mild symptoms, it would explain this discrepancy. Since this study involved the analysis of claims data, we were unable to confirm diagnosis or disease severity. The treatment of TD is highly variable based upon the severity of reactions, and duration of treatment could have been based on the severity of symptoms. It can be difficult to ascertain whether it was the treatment that truly impacted the outcomes or disease severity factored into health outcomes, as those with milder symptoms may not have required further treatment.

## LIMITATIONS

These results present with some limitations. For this study, we included only oral corticosteroid prescriptions and excluded prednisolone and dexamethasone. Topical corticosteroids are a common treatment for TD; however, for the purposes of this study, we focused on oral corticosteroids prescriptions. Further, we did not evaluate comorbidities, which could impact the duration of corticosteroids such as history of gastrointestinal bleeds or diabetes. Additionally, diagnoses were determined by healthcare insurance claims where some cases may have been missed or misdiagnosed and the primary reason for the follow-up visit could not be ascertained, which could have resulted in some misclassification error or selection bias. Also, claims data does not include years of experience or specific training of the clinician. As the ICD-10 diagnosis includes all plant-related ACD, a small portion of claims may have been for diagnoses other than poison ivy, oak, or sumac. Moreover, the reason of the return visit (eg, acute vs scheduled return) could not be obtained from the claims data. Lastly, the data only represented health claims from adults <65 who were privately insured and may not reflect trends in pediatric (<18 years old) or older adult (>65 years old) populations or those with different or no healthcare coverage.

## CONCLUSION

This study was the first to identify treatment patterns for toxicodendron dermatitis for those treated in the ED as well as explore the association between duration of treatment and healthcare utilization outcomes such as return visits. This research revealed that shorter duration oral corticosteroids and treatment received in the ED is associated with an increased risk for return healthcare visits. Most clinicians in the ED currently prescribe oral corticosteroids to TD patients for a duration of less than 14 days. Based on these results emergency physicians could reduce likelihood of ED return visit by adhering to recommendations of 14–21 days of treatment when medically appropriate.^6^ Future research should aim to structure interventions targeted at education on the appropriate treatment pathways for TD in order to reduce healthcare utilization associated with sub-therapeutic treatment.

## Figures and Tables

**Table 1:** Characteristics of claims 2017–2018 for toxicodendron dermatitis-related treatment.

Total number of eligible claims	115,885
Total unique adult patients	108,111 (93.29%)
Male	56,002 (51.80%)
Female	52,109 (48.20%)
Age (mean [SD]); [range]	44.19 (13.17); [18, 64]
Treatment locations first visit	
Emergency department	7,091 (6.56%)
Urgent care	10,554 (9.76%)
Dermatology	9,714 (8.99%)
Primary care*	47,719 (44.14%)
Other non-dermatology	33,033 (30.55%)
Total number of return claims (<28 days)	7,774 (6.71%)
Emergency department	470 (6.05%)
Urgent care	513 (6.60%)
Other	6,791 (87.36%)

* Primary care physicians include family and internal medicine clinicians.

*SD*, standard deviation.

**Table 2:** Frequency of oral corticosteroid prescriptions and duration of treatment at first visit 2017–2018.

	No prescription	1–13 Days	14–20 days	21+ days	Totals
Drug type					
No oral corticosteroid	60,637 (56.09)	-	-	-	60,637 (56.09)
Methylprednisolone	-	7,044 (99.52)	10 (0.14)	24 (0.34)	7,078 (6.62)
Prednisolone	-	10 (83.33)	2 (16.67)	0 (0.00)	12 (0.01)
Prednisone	-	30,315 (77.75)	7,650 (19.62)	1,025 (2.63)	38,990 (36.48)
Dexamethasone	-	152 (92.12)	10 (6.06)	3 (1.82)	165 (0.15)
Site/Specialty of first care					
Emergency department	3,194 (5.27)^	3,276 (86.26)	410 (10.80)	112 (2.95)	3,798 (8.21)*
Urgent care	4,714 (7.77)^	4,878 (85.37)	734 (12.85)	102 (1.79)	5,714 (12.36)*
Dermatology	7,847 (12.94)^	1,119 (61.99)	558 (30.91)	128 (7.09)	1,805 (3.90)*
Primary care	26,922 (44.40)^	17,534 (86.60)	2,325 (11.48)	387 (1.91)	20,246 (43.78)*
Other non-dermatologist	17,960 (29.62)^	10,714 (72.97)	3,645 (24.83)	323 (2.20)	14,682 (31.75)*
Total	60,637^	37,521 (81.14)	7,672 (16.59)	1,052 (2.27)	46,245 (100.00)*

^ Percentages for no prescription are based on the total number of visits in which patients received no oral corticosteroid prescription at the first visit.

* Totals are excluding no prescription counts.

**Table 3:** Univariable logistic regression: predictors of increased healthcare utilization 2017–2018

Outcome = return visit in 28 Days (n = 7,774)			

Predictor	Odds ratio	95% Wald confidence limits	P-value
Drug group (P <0.0001)			
No prescription vs prednisone	0.68	[0.65, 0.72]	<0.0001
Methylprednisolone vs prednisone	1.13	[1.02, 1.24]	0.01
Duration (P <0.0001)			
No prescription vs 14–20 days	0.84	[0.76, 0.93]	<0.001
1–13 days vs 14–20 days	1.32	[1.19, 1.46]	<0.0001
21+ days vs 14–20 days	1.20	[0.93, 1.55]	0.16
Initial treatment location (P <0.0001)			
Urgent care vs ED	0.83	[0.74, 0.93]	<0.001
Dermatologist vs ED	0.73	[0.65, 0.81]	<0.001
Primary care vs ED	0.77	[0.71, 0.85]	<0.001
Other non-dermatologist vs ED	0.78	[0.71, 0.86]	<0.001

**Outcome = Return Visit to the Emergency Department (n = 470)**			

Predictor	Odds ratio	95% Wald Confidence Limits	P-value

Drug group (P <0.0001)			
No prescription vs prednisone	0.65	[0.53, 0.80]	<0.001
Methylprednisolone vs prednisone	0.58	[0.40, 0.85]	0.005
Duration (P = 0.04)			
No prescription vs 14–20 days	0.69	[0.46, 1.02]	0.06
1–13 days vs 14–20 days	0.88	[0.60, 1.30]	0.51
21+ days vs 14–20 days	1.38	[0.59, 3.25]	0.46
Initial treatment location (P <0.0001)			
Urgent care vs ED	0.10	[0.07, 0.14]	<0.001
Dermatologist vs ED	0.03	[0.02, 0.06]	<0.001
Primary care vs ED	0.09	[0.07, 0.11]	<0.001
Other non-dermatologist vs ED	0.08	[0.06, 0.10]	<0.001

*ED*, emergency department.

**Table 4:** Multivariable logistic regression: predictors of increased healthcare utilization 2017–2018.

Outcome = return visit in 28 Days (n = 7,774)			

Predictor	Odds ratio	95% Wald Confidence Limits	P-value
Drug group (P = 0.001)			
No prescription vs prednisone	0.84	[0.76, 0.93]	0.001
Methylprednisolone vs prednisone	1.09	[0.99, 1.19]	0.09
Duration (P <0.0001)			
1–13 days vs 14–20 days	1.30	[1.17, 1.44]	<0.001
21+ days vs 14–20 days	1.17	[0.91, 1.51]	0.22
Initial treatment location (P <0.0001)			
Urgent care vs ED	0.82	[0.73, 0.92]	0.001
Dermatologist vs ED	0.92	[0.81, 1.04]	0.16
Primary care vs ED	0.80	[0.73, 0.88]	<0.001
Other non-dermatologist vs ED	0.81	[0.73, 0.90]	<0.001

*ED*, emergency department.
